# Physical Interactions Strengthen Chemical Gelatin Methacryloyl Gels

**DOI:** 10.3390/gels5010004

**Published:** 2019-01-17

**Authors:** Lisa Rebers, Tobias Granse, Günter E.M. Tovar, Alexander Southan, Kirsten Borchers

**Affiliations:** 1 Institute of Interfacial Process Engineering and Plasma Technology IGVP, University of Stuttgart, Nobelstr. 12, 70569 Stuttgart, Germany; lisa.rebers@igvp.uni-stuttgart.de (L.R.); tobias_granse@gmx.de (T.G.); alexander.southan@igvp.uni-stuttgart.de (A.S.); 2 Fraunhofer Institute for Interfacial Engineering and Biotechnology IGB, Nobelstr. 12, 70569 Stuttgart, Germany

**Keywords:** compression testing, physical and chemical network, hybrid network

## Abstract

Chemically cross-linkable gelatin methacryloyl (GM) derivatives are getting increasing attention regarding biomedical applications. Thus, thorough investigations are needed to achieve full understanding and control of the physico-chemical behavior of these promising biomaterials. We previously introduced gelatin methacryloyl acetyl (GMA) derivatives, which can be used to control physical network formation (solution viscosity, sol-gel transition) independently from chemical cross-linking by variation of the methacryloyl-to-acetyl ratio. It is known that temperature dependent physical network formation significantly influences the mechanical properties of chemically cross-linked GM hydrogels. We investigated the temperature sensitivity of GM derivatives with different degrees of modification (GM2, GM10), or similar degrees of modification but different methacryloyl contents (GM10, GM2A8). Rheological analysis showed that the low modified GM2 forms strong physical gels upon cooling while GM10 and GM2A8 form soft or no gels. Yet, compression testing revealed that all photo cross-linked GM(A) hydrogels were stronger if cooling was applied during hydrogel preparation. We suggest that the hydrophobic methacryloyl and acetyl residues disturb triple helix formation with increasing degree of modification, but additionally form hydrophobic structures, which facilitate chemical cross-linking.

## 1. Introduction

Gelatin is a collagen-derived biopolymer forming physical hydrogels due to the occurrence of secondary and tertiary structures and triple helix formation [1]. Physical gelling of the gelatin solutions is temperature dependent: Upon cooling, solutions gel at the gelation temperature and liquefy at the melting temperature [2]. Due to their inherent biocompatibility and bioactivity, gelatin-based hydrogels are frequently investigated for medical applications. For this purpose, hydrogels usually must be mechanically stable at body temperature, e.g., for drug release or tissue engineering [3,4], which is not fulfilled by physical gelatin hydrogels. Hence, thermally stable covalent cross-links are needed in gelatin hydrogels. This can be achieved for example with the use of carbodiimides, such as 1-ethyl-3-(3-dimethylaminopropyl)carbodiimide (EDC) [5] for cross-linking of unmodified gelatin, or by chemical modification of gelatin with cross-linkable groups such as methacryloyl groups, resulting in gelatin methacryloyl (GM, also known as GelMA) as originally introduced by van den Bulcke et al. [6].

GM is a gelatin derivative, which is widely investigated in the field of regenerative medicine [7,8]. It is usually synthesized by the reaction of methacrylic anhydride (MAAnh) with amino and hydroxyl functions of gelatin [9,10]. The molar ratio of the reactive groups of gelatin and applied MAAnh excess during synthesis adjusts the content of methacryloyl groups in the resulting GM [6,10,11,12]. The resulting methacrylamide and methacrylate functions can be radically cross-linked by application of a radical initiator. Typically, a photo-initiator (e.g., Irgacure 2959, or lithium phenyl-2,4,6-trimethylbenzoylphosphinate (LAP, Li-TPO-L)) is added and cross-linking is induced by UVA-irradiation. 

Increasing the degree of modification of gelatin generally results in a decrease of the physical interactions between the macromolecules [11,13,14,15] and thus results e.g., in lower solution viscosities and lower sol-gel transition temperatures. In our group, we provide GM derivatives with various, well defined amounts of reactive methacryloyl functions (e.g., GM2, GM5, GM10). Furthermore, we developed GM derivatives with additional acetyl functions, which are not reactive (GMA, e.g., GM2A8, GM5A5). The suffixes denote the molar excess of MAAnh or acetic anhydrate (AcAnh) which was applied during modification reaction, with regard to the amount of free amino groups in gelatin [6,10,11,12,15]. Thereby, it is possible to tune the chemical cross-linking potential, tunable by the degree of methacryloylation (DM), and the physical interaction potential, tunable by the total degree of modification, independently from each other.

Gelatin derivatives with a high degree of modification, e.g., generated by application of high amounts of MAAnh or AcAnh (GM10, GM2A8, GM5A5) [10,11,15], or by additional modification of the carboxylic functions of gelatin [14], show no physical gel formation at room temperature. These low viscous and non-gelling gelatin derivatives were described to be well suited for special 3D structuring methods such as inkjet-printing [15] and two photon polymerization [14,16].

On the other hand, the total degree of modification also influences the mechanical properties of chemically cross-linked GM hydrogels beyond the degree of methacryloylation: We observed recently that the storage moduli (*G′*) of chemically cross-linked GM hydrogels with low degrees of modification were unexpectedly high [11]. In particular, there was no difference detected between *G′* of chemically cross-linked GM2, GM5, and GM10 hydrogels. In contrast, *G′* increased as expected with increasing DM, if the total degree of modification was kept constantly high, as it is the case for GM2A8, GM5A5 and GM10. Based on these rheological data we assumed that GM2 and GM5 hydrogels were additionally stabilized by physical network formation due to their lower degree of modification compared to GM10, GM2A8, and GM5A5. These findings are supported by other studies, which took advantage of the physical gelling ability of GM hydrogel precursor solutions [13,14,17]: In these studies, compression moduli or storage moduli of GM hydrogels were increased dramatically, if physical network formation was first initiated by cooling of the GM solutions and UV-initiated chemical cross-linking was done afterwards. Similar effects were described for hydrogels out of unmodified gelatin that were cross-linked with enzymes [18,19,20] or a chemical cross-linker [21,22].

However, it is unknown thus far whether adequate thermal protocols can also be used to strengthen GM(A) hydrogels with high degrees of modification such as GM10 or GM2A8 which show no sol-gel transition above 10 °C due to strongly reduced physical interactions. In this study, we applied a cooling protocol to GM2, GM10 and GM2A8 hydrogel precursor solutions prior to chemical cross-linking and tested the compressive strength of resulting hydrogels. Since rheological data of GM(A) hydrogels are already heavily published, we chose compression testing as material characterization method. An advantage of this method is the possibility to apply higher stresses to the material compared to rheology. Thus, we developed an experimental set-up to determine the material response to compression at 37 °C in a swollen state.

## 2. Results and Discussion

**Figure 1:**
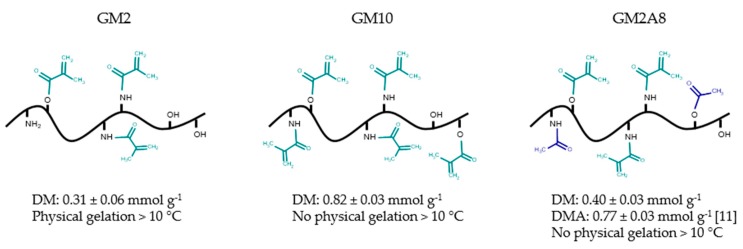
Schematic presentation of different gelatin derivatives used in this study. The suffix denotes the molar excess of anhydrides (MAAnh and AcAnh) used during synthesis procedure. Methacryloyl groups are marked green and acetyl groups blue. Furthermore, the degree of methacryloylation (DM), the total degree of modification (DMA, i.e., DM + degree of acetylation) and the capability to form physical gels [11] are given.

In this study, we used GMs with different chemical modification degrees: GM2, GM10, and GM2A8, see Figure 1. These derivatives were chosen because GM2 and GM2A8 have similar DMs (see Figure 1), but different total degrees of modification due to the acetylation of GM2A8 while GM10 and GM2A8 have similar total degrees of modification, but different DMs. 

Furthermore, two hydrogel preparation procedures were compared: Immediate chemical cross-linking of hydrogel precursor solutions after dissolution of the respective gelatin derivative at 37 °C (denoted with a “−“ in this study) and chemical cross-linking after cooling (20 min at 21 °C followed by 40 min at 4 °C) of the hydrogel precursor solution (denoted with a “+” in this study, also called sequential cross-linking in the literature). The chemical cross-linking was induced by 365 nm irradiation of the GM(A) solutions in alumina molds covered with quartz glass in presence of LAP as photo-initiator. All hydrogel precursor solutions were liquid in the molds when the photo-induced cross-linking was started immediately after transferring the warm solutions to the pre-warmed molds (−). GM2A8 were still liquid, when cross-linking was initiated after cooling during the sequential cross-linking procedure (+), as occasionally indicated by floating air bubbles. GM2 formed physical gels during the sequential cross-linking procedure.

**Figure 2:**
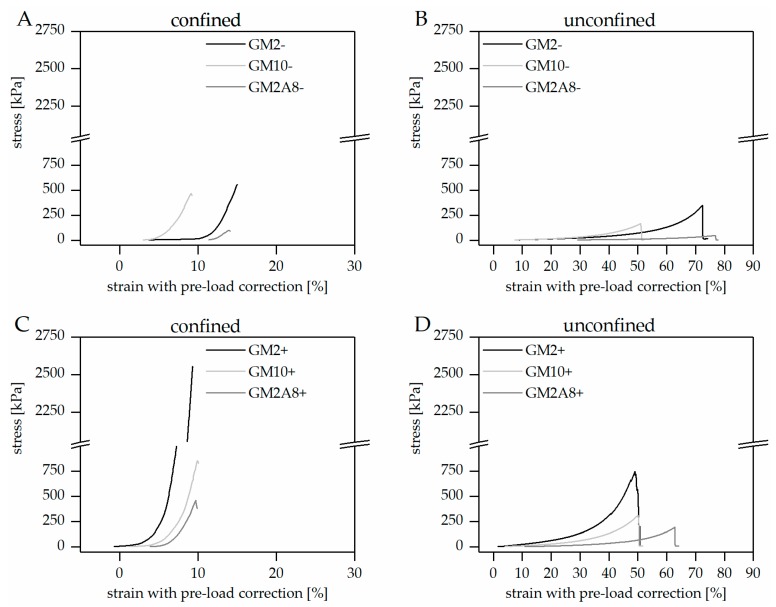
Stress-strain diagrams of gelatin methacryloyl acetyl (GM(A)) hydrogels made without (−) (**A,B**) and with cooling (+) (**C,D**) prior to chemical cross-linking and measured in confined (**A,C**) and unconfined (**B,D**) set-up. The utilized pre-load of 0.2 N deformed the gels to different extents; therefore we determined the respective deformation and added it to the measured strains, which shifted the curves to the right to the corrected, effectively applied overall strains, resulting in different initial strains for the various specimens. Material failure resulted in complete drop of stress in the unconfined set-up (**B,D**). In the confined set-up the first drop of stress was interpreted to indicate material failure and curves are only displayed up to the corresponding strain (**A,C**).

After chemical cross-linking, all hydrogels were tested in compression tests using a custom-made set-up for the measurement of hydrogels in a physiological setting (swollen state, phosphate buffered saline (PBS+), 37 °C), either in a confined or unconfined mode (see Section 4.5). Representative stress-strain diagrams for both measurement set-ups are shown in Figure 2. 

**Figure 3:**
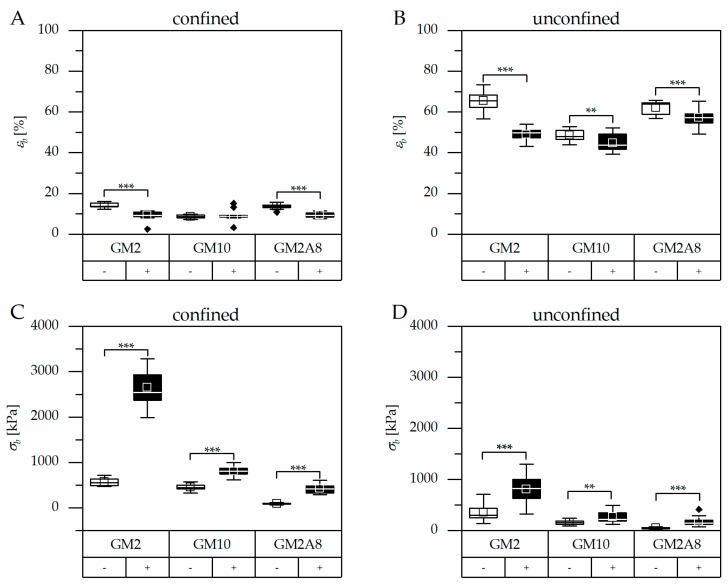
Compressive strains at break ($\varepsilon_{b}$) measured in the confined (**A**) or unconfined (**B**) set-up and the respective compressive stresses at break ($\sigma_{b}$) (**C**,**D**). Hydrogels were prepared with (+) or without (−) cooling. The 25th and 75th percentiles of all specimen determine the boxes. The whiskers mark the inner fence (1.5-fold interquartile range). Data points beyond the inner fence are outliers and shown as black diamonds. The line in the box represents the median and the center of the square the mean. Significant differences are marked as follows: ** *p* < 0.01, *** *p* < 0.001.

The stress-strain curves of all hydrogels were generally of a comparable shape, regardless of the hydrogel preparation procedure and the measurement set-up: The hydrogels showed relatively low stress responses at low strains, resulting in low slopes. The slopes generally increased upon increasing deformation indicating strain stiffening of the hydrogels until material failure. Similar behavior has been described for hydrogels before [23,24]. It has to be noted that the pre-load of 0.2 N, which was applied to avoid artefacts due to incomplete contact of the indenter with the specimens, resulted in relevant deformations especially of the softer hydrogels. This initial compression was taken into account and added to the strains applied during the measurement (see Figure 2 and Section 4.6). The compressive strains at break ($\varepsilon_{b}$) and the respective compressive stresses at break ($\sigma_{b}$) were extracted from such corrected stress-strain curves (Figure 3, Table 1). 

Hydrogels are not able to extend laterally in the confined measuring set-up. Therefore, the effective vertical strains were generally smaller compared to the unconfined set-up, in particular the compressive strains at break ($\varepsilon_{b}$). Correspondingly, the stress and in particular the compressive strength at break ($\sigma_{b}$) was greater in the confined set-up than in the unconfined set-up. Furthermore, the relative impact of cooling on $\varepsilon_{b}$ and $\sigma_{b}$ was also different in the confined and unconfined measurement set-ups (e.g., $\sigma_{b}\;($GM2(+))/$\sigma_{b}\;($GM2(−)) = 4.6 in the confined measurement, or $\sigma_{b}\;($GM2(+))/$\;\sigma_{b}\;($GM2(−) = 2.2 in the unconfined measurement; $\sigma_{b}\;($GM10(+))/$\sigma_{b}\;($GM10(−)) = 1.8 in the confined measurement, or $\sigma_{b}\;($GM10(+))/$\sigma_{b}\;($GM10(−)) = 1.6 in the unconfined measurement). This makes it obvious that data from the confined and unconfined measurements must not be mixed up for comparison, not even for relative correlation. Consequently, standardized procedures for hydrogel characterization are required in order to enable generation and publication of comprehensive and informative data sets which can then be compared across laboratory borders.

Firstly, we consider the hydrogels which were prepared without cooling: $\varepsilon_{b}$ of GM2A8(−) was significantly higher than that of GM10(−), and simultaneously $\sigma_{b}$ of GM2A8(−) was lower than that of GM10(−), as it was expected due to the differences in the DM. In contrast, the compressive properties of GM2(−) were exceptional. GM2(−) gels were as compressible as GM2A8(−) gels, but simultaneously the compressive strength at break of GM2(−) was higher than GM2A8(−) and even higher than GM10(−). This confirmed our prior observations from rheological characterizations, that GM2A8 hydrogels had a lower *G′* compared to GM10 hydrogels, and that *G′* of GM2(−) hydrogels were also significantly higher compared to GM10 and GM2A8 hydrogels [11]. 

The lower storage modulus *G′* and compressive strength $\sigma_{b}$ of GM10(−) and GM2A8(−) compared to GM2(−) can be assigned to the known effect that elevated degrees of modification restrain the ability of GM materials to form triple helices [14,15]. It can be derived from the presented data that it was mainly the different amounts of chemical bonds of GM2A8(−) and GM10(−) which defined the compressive strengths of such hydrogels, while physical network formation additionally contributed to strengthening of GM2(−). This was the case, although none of the hydrogel precursor solutions was cooled below the gelation temperature *T*_gel_ but all were cross-linked in the liquid state. Obviously, some beneficial presorting of the GM2 took place, although gelling was not visually observed.

As a consequence, we wondered whether cooling of the hydrogel precursor solutions could also induce strengthening of hydrogels made of highly modified GM(A)s, which do not show gel transition above 10 °C. Therefore, we used a cooling procedure (20 min at 21 °C followed by 40 min at 4 °C) prior to chemical cross-linking to impose temperature-dependent physical interactions. The results are shown in Figure 2 and Figure 3 graphically. The measured $\sigma_{b}$ and $\varepsilon_{b}$ are given in Table 1 for an easier access. The compressibility of all (+) hydrogels was significantly reduced by cooling, i.e., hydrogels were less compressible (Figure 3A/B, *p* < 0.01; except for GM10 hydrogels measured in the confined set-up where $\varepsilon_{b}$ remained unchanged). Simultaneously, the $\sigma_{b}$ values increased significantly for all (+) hydrogels (*p* < 0.01, Figure 3C/D). 

This was expected for GM2(+) gels, because GM2 solutions obviously formed physical hydrogels upon cooling. Physical gelling has been correlated with triple helix formation of gelatin and GM derivatives in several studies [13,14,17]. It was suggested that these quaternary structures are then fixed by covalent cross-links such that both, physical and chemical networks contribute to the elastic stability of the hydrogel and temperature-related melting was hindered [11,13,17]. It is discussed that the helices could act as templates for a more effective cross-linking of the C=C double bonds of gelatin methacryloyl. Indeed, unreacted C=C double bonds were still detectable by Fourier transform infrared spectroscopy of GM gels that were UV-cured at 37 °C, but not in gels made from the same material after incubation at 4 °C to allow physical gelling before UV-curing [13].

Additionally, the presented results in this study show that strengthening by cooling was also effective in GM2A8(+) and GM10(+) hydrogels with high degrees of modification. This was rather unexpected, as we proofed in earlier studies that physical interactions are strongly reduced in GM2A8 and GM10 solutions, resulting e.g., in decreased viscosity and absent physical gelation for temperatures above 10 °C [11]. Therefore, we further investigated GM2, GM10, and GM2A8 hydrogel precursor solutions rheologically during the thermal protocol in order to learn more about the gelation process during cooling (see Section 4.7, Table 2, and Figure S1 in the supporting information). GM2 solutions gelled (i.e., *G′ ≥ G″*) after 3 min at 21 °C as expected. Additionally, we detected *G′ ≥ G″* for GM10 solutions after approx. 4 min at 4 °C. GM2A8 solutions showed gelling just for one out of three batches after 18 min at 4 °C, while no gelling was observed at all during 40 min at 4 °C for two out of three batches. At the end of the thermal protocol *G′* of physical GM2 gels was the highest (10,633 ± 514 Pa, *n* = 3), *G′* of GM10 gels was lower (2112 ± 1264 Pa, *n* = 3) than that, and *G′* of gelled GM2A8 was extremely low (24 Pa, *n* = 1). 

The ratio of *G″* and *G′* (loss factors tan(δ), see Table 2) of the cooled solutions accentuate the *G′* data: tan(δ) of GM2A8 (0.165, single measurement) was higher than tan(δ) of GM10 (~0.013) and GM2 (~0.008). The approx. 100 times higher *G*′ than *G*″ points out that GM2 and GM10 both formed networks with elastic properties, although the physical GM2 gels were much stronger than physical GM10 gels, as revealed by the absolute values of *G′*. Physical GM2A8 gels showed a rather viscous behavior. This was confirmed by the fact, that just one out of three measured hydrogel precursor solutions showed a gelation point. 

If the formation of strong physical GM2(+) gels was due to the formation of triple-helical regions, conversely, the low or very low *G′* suggested reduced or no helix formation in GM10(+) and GM2A8(+) solutions. In native triple helices 30 amino acids per strand are associated in one triple helix turn [25]. We calculated the theoretical mean distance between two methacryloyl- or acetyl-modified amino acids in the GM(A) derivatives as the reciprocal of the total degree of modification (see Table S1). We assumed an average molecular weight of amino acids (based on the amino acid composition of the raw material published in [11]) of 122 g mol^−1^ to calculate the theoretical number of amino acids between two chemical modifications. According to this estimation, one chemically modified amino acid occurs statistically every 26 amino acids in GM2, while in GM10 and GM2A8 one modification occurs every 10 amino acids. Thus, theoretically in GM2 one amino acid per triple helix turn is chemically modified, while in GM10 and GM2A8 there are three modified amino acid per triple helix turn. In fact, GM2 is mainly modified at lysine residues [10,11]. Regarding the lysine distribution in the triple-helical region of the collagen α-chain (bovine collagen type I, according to Uniprot database), 83% of lysines in this region are separated by more than 30 other (not lysine) amino acids. Thus it seems plausible, that triple-helix formation and thus significant stiffening of the gels can occur in GM2 solutions, but not in GM10 and GM2A8 solutions, since in GM10 and GM2A8 other hydroxyl containing amino acids are modified additionally.

In this context we want to consider additional factors which could be responsible for the strengthening of the GM10(+) and GM2A8(+) hydrogel network besides the assembly of triple helices, i.e., re-arrangement of GM(A) polymer chains by hydrophobic interactions of methacryloyl and acetyl groups. Methacryloyl and acetyl groups are rather hydrophobic and might form hydrophobic domains in an aqueous environment. Thereby, a polymer network with few open loops might form and the hydrophobic methacryloyl groups might be oriented in favor of higher conversions during chemical cross-linking. Cooling would help to stabilize such hydrophobic domains and consequently the density of elastically active chemical cross-links would be enhanced compared to solutions that are cross-linked at higher temperatures. In this way, the insertion of methacryloyl and acetyl groups might have a structure-forming effect apart from hindering the formation of triple-helices. 

Indeed, we observed before that the hydrodynamic radius of gelatin derivatives decreased with increasing degrees of modification [11]. We assigned this to the masking of the hydroxyl groups of hydoxyproline in the amino acid sequence by insertion of hydrophobic methacryloyl or acetyl-residues. The resulting loss of hydrogen bindings can be expected to de-stabilize the native, elongated α-helix conformation of single collagen or gelatin molecules, and to promote the (more globular) random coil conformations with shielded hydrophobic domains to exclude water. The hydrodynamic radius was even smaller in derivatives with higher acetyl portions, possibly because less volume was needed by acetyl than by methacryloyl-groups [11]. We hypothesize an impact of the type and size of the inserted methacryloyl and acetyl functions: Finally, such differences in the hydrodynamic radius might influence the degree of entanglement of GM(A) molecules and account for the unexpected rheological difference of GM10 and GM2A8 (Table 2) in spite of the very similar degrees of modification of the two derivatives. 

To summarize, we suggest that hydrophobic interactions contribute to the formation of dense protein domains and by that to physical networks in gelatin methacryloyl hydrogels. Cooling stabilizes such hydrophobic domains and thus radical cross-linking of the methacryloyl groups becomes more effective resulting in stronger hydrogels at lower temperatures. In gelatin derivatives with low degrees of modification the formation of triple helices occurs in parallel to formation of hydrophobic domains. Thus the resulting hydrogels are stronger than hydrogels based on gelatin derivatives with a high degree of modification, where hydrophobic domains are the dominant structure while formation of stiff triple helical regions is hindered. 

This hypothesis is supported by significant differences in the temperature sensitivity of cross-linked GM(A) hydrogels and hydrogels which are generated by chemical cross-linking of unmodified gelatin with a chemical cross-linker [21] or enzymatically [18]: Other authors [13,17] and ourselves [11] have observed that the mechanical properties of chemically cross-linked GM(A) hydrogels were not temperature-dependent, no matter whether they had been cross-linked in the gelled or in the liquid state. The compression tests in this study were conducted at 37 °C. Although this is above the melting temperature for all GM(A)s [11], the mechanical differences between gels that had been cross-linked in the liquid state or the (quasi-)gelled state were conserved: The interactions that additionally strengthened the hydrogels were not lost upon heating. This indicates that in the macromolecular network of chemically cross-linked GM(A) hydrogels triple helices and other physical interactions neither form nor melt. In contrast, authors who performed cross-linking of unmodified gelatin hydrogels with bis(vinylsulfonyl)methane [21,22] observed temperature-sensitivity of the hydrogels: Helices were reversibly formed by a temperature switch after the chemical cross-linking reaction had been stopped. The same was observed for enzyme catalyzed cross-linking of gelatin with transglutaminase. We postulate that the superimposed hydrophobic structure, which is then fixed by covalent cross-linking, might be the reason for the distinct temperature stability of GM gels, which is not fully achieved by chemical cross-linking of unmodified gelatin. Consequently, the hydrophobic nature of the inserted methacryloyl-functions and acetyl-functions enables formation of gelatin hydrogels with elevated strength.

## 3. Conclusions

The compressive strength of chemically cross-linked gelatin methacryloyl gels can be increased by cooling whether physical gel formation is visually observed or not. This indicated that temperature dependent physical interactions can be used to strengthen cross-linked GM(A) hydrogels, e.g., hydrophobic interactions in the case of highly modified gelatin derivatives. On the other hand, it also emphasizes the need to tightly control the thermal history of GM(A) solutions even if physical gelation is not an obvious issue. Furthermore, quaternary structures based on hydrophobic interactions may facilitate the radical cross-linking of GM(A) and result in hydrogels, which are temperature independent and stable at 37 °C, other than enzymatic or chemical cross-linking of unmodified gelatin. This can make a relevant difference regarding possible biomedical application. 

## 4. Materials and Methods

### 4.1. Materials

The following materials were purchased from Sigma Aldrich (Darmstadt, Germany): Acetic anhydride (AcAnh), sodium hydrogen phosphate (Na_2_HPO_4_), Dulbecco’s phosphate buffered saline with MgCl_2_ and CaCl_2_ (PBS+), methacrylic anhydride (MAAnh) as well as sodium hydroxide (NaOH). Sodium 3-trimethylsilyl-propionate-2,2,3,3-d4 (TMSP) was bought from Merck (Darmstadt, Germany). Other reagents were purchased from the following sources (given in parentheses): Deuterium oxide (D_2_O) (Deutero; Kastellaun, Germany), Gelatin (type B, Limed, bovine bone, 232 Bloom, standard viscosity = 4.5 mPa·s; Gelita; Eberbach, Germany). Dialysis membranes (MWCO 12 kDa–14 kDa) were purchased from Medicell International Ltd. (London, UK). The photoinitiator lithium phenyl-2,4,6-trimethylbenzoylphosphinate (LAP, also known as Li-TPO-L, TMPPL) was synthesized according to Fairbanks et al. [26]. ^1^H-NMR spectroscopy (500 MHz, D_2_O, d) proofed adequate shifts and integrals, i.e., 7.74 (m, 2H), 7.56 (m, 1H), 7.46 (m, 2H), 6.87 (2H, s), 2.22 (s, 3H), 2.04 (s, 6H). 

### 4.2. Synthesis of Gelatin Methacryloyl (GM2, GM10) and Gelatin Methacryloyl Acetyl (GM2A8)

Gelatin methacryloyl (GM) was prepared as previously described [10]. In short, gelatin (25.01 g) was dissolved in deionized water (250 mL) at 37 °C and its pH was adjusted to 7.3 with the use of an automatic titration device. Within 30 min 2.70 g/13.49 g of MAAnh were added, which corresponds to a two-fold (GM2) or ten-fold (GM10) molar excess relative to an amino group content of gelatin (0.35 mmol g^−1^ [6]). In case of GM2A8 synthesis, after 2 h of methacryloylation reaction 7.15 g of acetic anhydride (AcAnh) were added dropwise within 30 min, resulting in an eight-fold (GM2A8) molar excess relative to the content of amino-groups of gelatin [6]. The reaction mixture was stirred vigorously for 5 h in total, keeping its pH constantly between 7.0 and 7.4. The reaction mixture was filtrated subsequently and its pH was adjusted to 9.5. After leaving the mixture at 4 °C for 2 days, the solution was dialyzed for 4 days against deionized water at room temperature. Afterwards, the pH was adjusted to 8.5 and the solution was freeze-dried. The degree of methacryloylation (DM) was determined using ^1^H-NMR spectroscopy as described by Claaßen et al. [10]. The mean and the standard deviation of the three syntheses of each derivative used in this study are given in Figure 1. 

### 4.3. Preparation of GM(A) Hydrogels

Hydrogels with initial GM(A) concentration of 10% (*w*/*w*) were prepared either by immediate photo-initiated radical cross-linking or by cooling of the precursor solution and subsequent chemical cross-linking. In detail, GM(A) was dissolved in PBS+ containing 0.2% (*w*/*w*) LAP with regard to biopolymer content for 2 h at 37 °C. Afterwards, the solution was poured into a cylindrical mold (25 mm diameter, 5 mm depth) and covered by a quartz glass pane. If physical gelation of hydrogel precursor solution was desired, the mold was left at 21 °C for 20 min and afterwards for 40 min at 4 °C. Then, chemical cross-linking was done by exposure to UVA light (approx. 365 nm, 18.2 mW cm^−2^) for 2.5 min. If physical gelation was not desired, the hydrogel precursor solution was cured immediately after pouring into the mold. After curing, the quartz glass pane was removed and the cross-linked hydrogels were taken out of the mold. Hydrogels were washed for 5 h in PBS+ at 37 °C, exchanging the PBS+ every hour. Afterwards, the hydrogels were swollen in PBS+ for 19 h at 37 °C. 

### 4.4. Measurement Set-Up for Hydrogel Compression Testing

**Figure 4:**
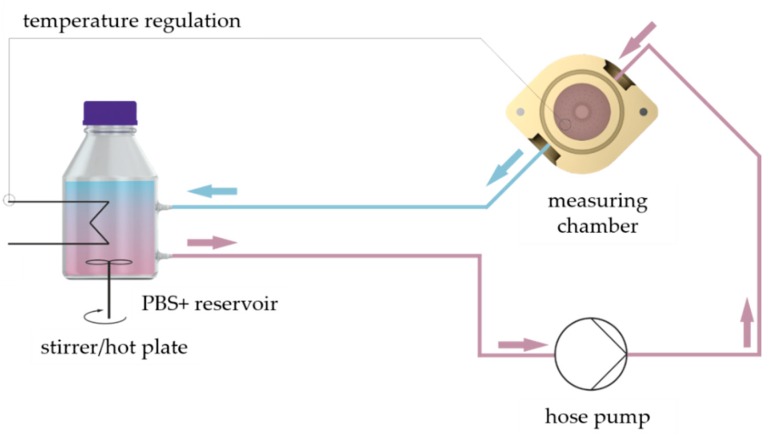
Experimental set-up for compression tests of hydrogels in a swollen state at 37 °C. The measuring chamber was flushed with 37 °C warm PBS+ throughout the measurements. The PBS+ was warmed on a magnetic stirrer/hotplate and pumped in a closed circuit through the measuring chamber by the hose pump.

We used the material testing machine Allround-Line Z005 equipped with a 2.5 kN Xforce HP load cell (Zwick, Ulm, Germany) for compression testing of GM(A) hydrogels. The appropriate software “testXpert II” was utilized for execution of the measurements and analysis of the data. Furthermore, a measurement set-up was established to enable measurement of hydrogels at 37 °C in a swollen state (Figure 4): A measuring chamber made from polyether ether ketone (PEEK) was used with an inflow and outflow. A hose pump pumped PBS+ through the measuring chamber in a closed circuit. The PBS+ was warmed to 37 °C on a stirrer/hotplate, which was controlled by a temperature sensor in the chamber. 

**Figure 5:**
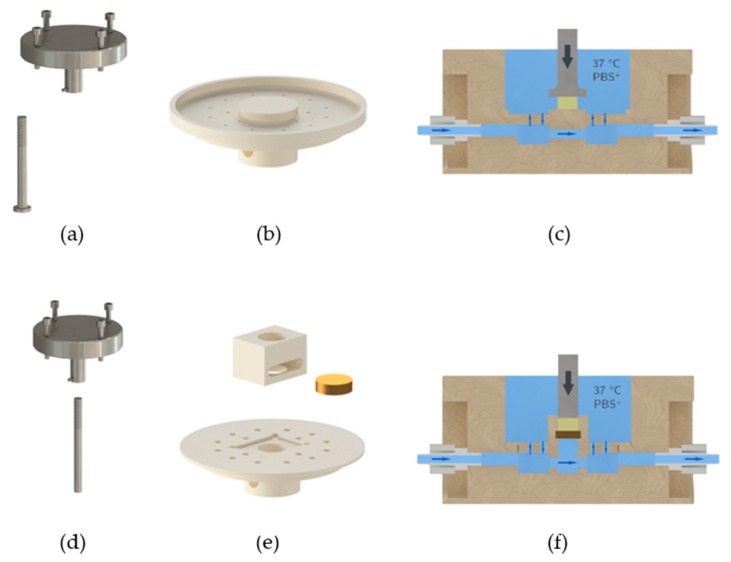
Technical drawing of the measurement components for unconfined (**a**–**c**) and confined (**d**–**f**) measurements: (**a**/**d**) Self-designed measuring head for the material testing machine Z005, consisting of an adapter and the measuring head. The diameter was 15 mm (**a**) and 8 mm (**d**). (**b/e**) Technical drawing of the inlet of the measuring chamber. Due to the perforation of the inlet the exchange of the PBS+ surrounding the hydrogel was ensured. For confined testing a chamber with a filter disk ensuring PBS+ exchange was utilized. (**c/f**) Transverse section of the measuring chamber.

To ensure PBS+ exchange within the measuring chamber without uncontrolled movement of the hydrogel we constructed a perforated inlet with a platform, see Figure 5b,c. The hydrogels were placed on that platform for measurements and were covered completely with PBS+. We constructed an adapter for the Z005 tool holder of the testing machine and a measuring head with a diameter of 15 mm, see Figure 5a. The adapter was fixed at the Z005 tool holder using four M5 screws. The measuring head was screwed into the adapter by its M8 thread. A M3 grub screw was used to cinch the screwed measuring head.

### 4.5. Compression Testing of GM(A) Hydrogels

Cylindrical hydrogel samples (ø 8 mm) were punched out of the washed and swollen hydrogel in quadruplicates for compression testing. Samples were stored in 37 °C warm PBS+ until the measurement. The geometry of the hydrogel samples is crucial for the output data, since for calculation a perfect cylindrical sample shape is assumed. Therefore, the height of every sample before compression testing was determined with a caliper. 

Failure testing of hydrogels was performed at 37 °C in a swollen state using a pre-load of 0.2 N and 0.5% s^−1^ as test speed. For unconfined measurements, the hydrogel was placed into the measurement chamber without any lateral limitation and a measuring head with a diameter of 15 mm was used. A compression chamber with a filter disk as bottom and a standard indenter with a diameter of 8 mm were utilized for confined measurements.

For the measurements, the hydrogel sample was placed in the middle of the inlet platform and the measuring head was positioned 5.5 mm above the inlet of the measuring chamber. After 4 min waiting in this position, the measuring head sloped with 0.1 mm s^−1^ to the hydrogel sample until a pre-load of 0.2 N was achieved. After reaching this pre-load, the height of the test specimens was recorded ($h_{0.2\;N}$) and the measurement started automatically with a constant strain rate of 0.5% s^−1^ relative to the initial test specimen’s height until destruction. 

### 4.6. Data Analysis of Compression Testings

The stress–strain relationship resulting out of compression testing was analyzed. Since hydrogels have a progressive stress–strain relationship like elastomers, the compression strength and compressive stress at break were the same and we named it $\sigma_{b}$. The compressive strain at break or compressive strainat compressive strength is named $\varepsilon_{b}$ in this study. 

We programmed the software “testXpert II” to register the compressive strength at break ($\sigma_{b}^{reg}$) and the compressive strain at break ($\varepsilon_{b}^{reg}$) in confined measurements automatically after a strength drop of 50 kPa. In unconfined measurements the maximum registered stress was defined to be $\sigma_{b}$, the corresponding compressive strain was defined to be $\varepsilon_{b}^{reg}$.

Furthermore, we recognized a significantly reduced height ($h_{0.2\;N})$ for soft gels at the chosen pre-load of 0.2 N. Consequently, we had to correct the registered values for the compressive strains at break $\varepsilon_{b}^{reg}$, because the machine regarded $h_{0.2\;N}$ as reference height instead of the original heights of the gels $h_{0}$. We measured the original heights of hydrogels ($h_{0}$) manually with a caliper. The corrected values for ε were then calculated using Equation 1. Specifically, the height at break $h_{b}$ was determined as the measured height at $\sigma_{b}^{reg}$. The corrected $\varepsilon_{b}$ was calculated using Equation 2. 

(1) $$\varepsilon =\frac{\left(h_{0}-h\right)}{h_{0}}\cdot 100\%$$

(2) $$\varepsilon_{b}=\frac{\left(h_{0}-h_{b}\right)}{h_{0}}\cdot 100\%$$

### 4.7. Temperature Depending Rheological Measurements

A Physica Modular Compact MCR301 rheometer from Anton Paar (Ostfildern, Germany) equipped with cone-plate system (*d* = 40 mm) was used to investigate *G′* and *G″* of the hydrogel precursor solutions (see Section 4.3) as a function of temperature. *G′* and *G″* were determined at a fixed amplitude (5%) and frequency (1 s^−1^). 

To reproduce the utilized gelation procedure, we performed the following temperature changes during rheological measurements: The 37 °C warm hydrogel precursor solution was put into the measuring gap and the measurement was started. The first 5 min the temperature was 37 °C, afterwards the bottom plate of the rheometer was cooled to 21 °C for 20 min followed by a further cooling to 4 °C for 40 min. *T*_gel_ was determined from the cooling curve *G′* = *G″*. Since usually the temperature where *G′* = *G″* is not measured exactly, the first temperature where *G′* > *G″* (cooling curve, *T*_gel_) was used.

### 4.8. Statistics and Box-Plots

The presented data include hydrogels out of three independent synthesis per gelatin derivative. Furthermore, we did compression testing on quadruples of each hydrogel. For statistical analysis, a *t*-test was utilized. We chose modified box-plots for data presentation. The boxes represent 75% of the data; the line within the box marks the mean and the square the median. Whiskers tag the 1.5-fold interquartile range, statistical outliers are represented as hash outside the whiskers.

## Figures and Tables

**Table 1:** Compressive strain at break ($\varepsilon_{b}$) and the respective compressive stresses at break ($\sigma_{b}$) of GM(A) hydrogels measured in a confined or an unconfined setting. Each GM(A) hydrogel was investigated without cooling (−) and with cooling (+) before chemical cross-linking.

Gelatin Derivative		Confined		Unconfined	
		*σ_b_* [kPa]	*ε*_*b*_ [%]	*σ_b_* [kPa]	*ε*_*b*_ [%]
GM2	−	570.6 ± 31.6	14.0 ± 0.4	358.6 ± 117.4	65.4 ± 4.0
	+	2647.7 ± 42.9	9.2 ± 2.1	805.8 ± 96.3	48.9 ± 0.6
GM10	−	457.2 ± 24.9	8.7 ± 0.6	159.4 ± 45.7	48.5 ± 2.8
	+	801.4 ± 67.5	9.2 ± 1.1	251.6 ± 59.2	44.8 ± 2.6
GM2A8	−	91.0 ± 8.6	13.7 ± 0.9	47.3 ± 8.6	62.1 ± 1.4
	+	423.3 ± 56.6	9.5 ± 0.7	180.9 ± 21.7	57.1 ± 1.5

**Table 2:** Rheological analysis of GM(A) solutions (10% (*w*/*w*)) during cooling (20 min 21 °C + 40 min 4 °C). Gelation was determined when the storage modulus *G′* became larger than the loss modulus *G´´* (*G′ ≥ G″*). Furthermore, the storage moduli of the physical hydrogels measured at the end of the thermal protocol (after 60 min), before chemical cross-linking, is given (*G′*). The loss factor of physical hydrogels is determined as ratio of *G″* and *G′* (tanδ).

	GM2 (*n* = 3)	GM10 (*n* = 3)	GM2A8 *
G′ ≥ G″	3 min/21 °C	20 min/21 °C + 4 min/4 °C	20 min/21 °C + 18 min/4 °C
*G′* [Pa]	10,633 ± 514	2112 ± 1264	48
tan(δ) (G″/G′)	0.008 ± 0.000	0.013 ± 0.003	0.165

* To be noted: A gelation point *G′ ≥ G″* was singularly detected for one batch out of three, the other two solutions showed no sign of gelling, therefore no standard deviations were determined.

## Supplementary Materials

The following additional data are available online at http://www.mdpi.com/2310-2861/5/1/4/s1. Figure S1. Rheological evaluation of GM(A) gelling behavior during the chosen thermal protocol: (A) Storage moduli (*G′*) and (B) loss moduli (*G″*). Table S1. Degree of modification of gelatin derivatives.
