# Supplementary material for: Physical Interactions Strengthen Chemical Gelatin Methacryloyl Gels
Source: Gels. 2019 Jan 17;5(1):4. doi: 10.3390/gels5010004 (PMC6473781; doi:10.3390/gels5010004)
Supplement: Supplementary file 1 [file gels-05-00004-s001.pdf]

Supporting Information

Physical Interactions Strengthen Chemical Gelatin Metacryloyl Gels

Lisa Rebers[1], Tobias Granse[1,2], Günter E. M. Tovar [1,2,*], Alexander Southan[1] and Kirsten Borchers[1,2,*]

[1] Institute of Interfacial Process Engineering and Plasma Technology IGVP, University of Stuttgart; Nobelstr. 12, 70569 Stuttgart, Germany; lisa.rebers@igvp.uni-stuttgart.de (L.R.); tobias_granse@gmx.de (T.G.); guenter.tovar@igvp.uni-stuttgart.de (G.E.M.T); alexander.southan@igvp.uni.-stuttgart.de (A.S.)

[2] Fraunhofer Institute for Interfacial Engineering and Biotechnology IGB; Nobelstr. 12, 70569 Stuttgart, Germany

* Correspondence: kirsten.borchers@igb.fraunhofer.de; Tel.: +49-(0)711-970-4121, guenter.tovar@igvp.uni-stuttgart.de; Tel.: +49-(0)711-970-4109

Table S1. Degree of modification of gelatin derivatives. The mean distance of two chemically modified amino acids was calculated as the reciprocal of the degree of modification. The amount of amino acids between the two chemically modified amino acids was estimated assuming an average molecular weight of amino acids of 122 g mol^{-1} calculated out of the amino acid composition of the used gelatin type B (composition can be found in [11]).

	Degree of Modification [mmol g^{-1}]	Mean distance between two chemical modified amino acids [g mol^{-1}]	Mean number of amino acids between two chemical modified amino acids
GM2	0.31	3158	26
GM10	0.82	1194	10
GM2A8	0.77	1271	10

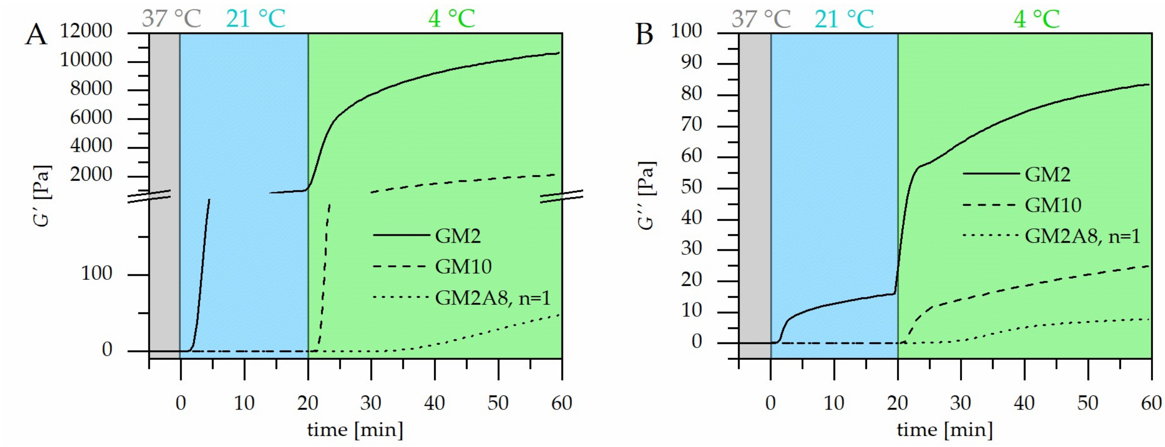

Figure S1. Rheological evaluation of GM(A) gelling behavior during the chosen thermal protocol: (**A**) Storage moduli (*G′*) and (**B**) loss moduli (*G′′*). Note the interruption of the y-axis the associated change of scales. Due to this interruption the curves of GM10 and GM2A8 both end at 200 Pa and start again at 800 Pa.
